# Editorial: Exploring neuropsychiatric disorders through multimodal MRI: network analysis, biomarker discovery, and clinical insights

**DOI:** 10.3389/fnins.2026.1960342

**Published:** 2026-09-18

**Authors:** Ruichen Ren, Wei Wang

**Affiliations:** 1 Henan Clinical Research Center of Infectious Diseases (AIDS), Affiliated Infectious Diseases Hospital of Zhengzhou University, Henan Infectious Diseases Hospital, Zhengzhou, Henan, China; 2 Department of Radiology, Beijing Youan Hospital, Capital Medical University, Beijing, China

**Keywords:** clinical insights, multimodal MRI, network analysis, neurological disorders, neuropsychiatric disorders

Magnetic resonance imaging (MRI) has become an essential tool for investigating the complex pathophysiological mechanisms underlying neuropsychiatric and neurological disorders. In contrast to conventional approaches that rely on a single imaging measure, multimodal MRI integrates information on brain structure, tissue microstructure, functional activity, and dynamic network organization, thereby providing a multidimensional characterization of disease-related brain alterations. In highly heterogeneous brain disorders, similar clinical manifestations do not necessarily arise from the same biological substrates, and subtle alterations in brain networks may emerge before clinically apparent functional impairment. Accordingly, the integration of multiple imaging modalities, characterization of brain network organization, and incorporation of clinical and biological information have become important strategies for identifying imaging biomarkers and advancing precision phenotyping. This Research Topic focuses on the application of multimodal MRI to neuropsychiatric and neurological disorders, with particular emphasis on structure–function integration, dynamic brain activity, disease heterogeneity, and the integration of imaging findings with clinical and molecular information. Collectively, these studies aim to advance imaging biomarkers from initial discovery to validation and clinical translation.

The research included in this Research Topic span a broad range of conditions, including neurodevelopmental disorders, psychiatric disorders, neurodegenerative diseases, infection-related brain injury, pain-related disorders, disorders of consciousness, and spinal disorders affecting the nervous system. Despite the diversity of the conditions investigated, these studies share a common goal: to use multimodal neuroimaging to characterize disease-related structural abnormalities and functional reorganization, and to determine how these alterations relate to clinical and biological measures. Together, they provide new evidence for understanding the heterogeneity of brain disorders and identifying candidate imaging biomarkers (Figure 1).

**Figure 1:**
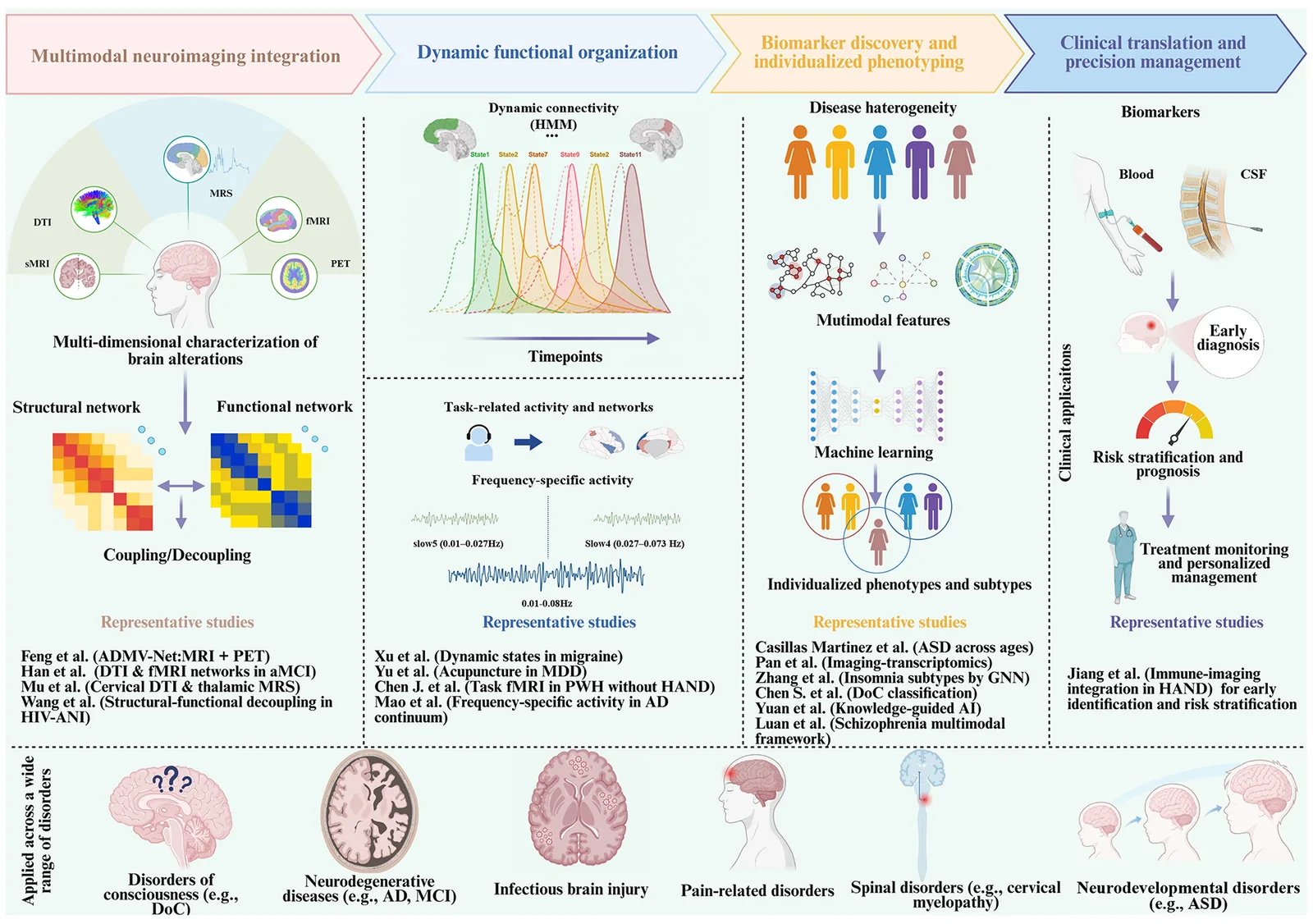
Conceptual framework for multimodal neuroimaging in neuropsychiatric and neurological disorders. The framework summarizes four interrelated directions represented in this Research Topic. First, multimodal neuroimaging integrates structural MRI (sMRI), diffusion tensor imaging (DTI), functional MRI (fMRI), positron emission tomography (PET), and magnetic resonance spectroscopy (MRS) to characterize disease-related brain alterations and structure–function relationships. Second, dynamic connectivity, task-related activity, and frequency-specific analyses capture temporal and context-dependent functional organization. Third, multimodal features combined with machine learning facilitate the investigation of disease heterogeneity and individualized phenotypes. Fourth, integration of neuroimaging with peripheral blood and cerebrospinal fluid biomarkers may support early diagnosis, risk stratification, prognosis, treatment monitoring, and personalized management. The lower panel illustrates applications across neurodevelopmental, psychiatric, neurodegenerative, infection-related, pain-related, consciousness, and spinal disorders.

## Multimodal fusion and structural-functional coupling reveal multi-level brain alterations

Single MRI modality typically captures only a subset of disease-related abnormalities, whereas multimodal imaging fusion provides a framework for integrating structural, microstructural, and functional information. Feng et al. developed ADMV-Net, which integrates multiview features from MRI and positron emission tomography (PET) to classify individuals with Alzheimer's disease, mild cognitive impairment, and normal cognition, thereby demonstrating the potential of multimodal fusion models for disease identification. Han et al. compared structural networks derived from diffusion tensor imaging (DTI) with functional networks derived from resting-state functional MRI (rs-fMRI) in patients with amnestic mild cognitive impairment and found distinct patterns of structural disruption and functional reorganization, suggesting the presence of cross-modal network alterations at an early stage of cognitive impairment. In a study of HIV-associated asymptomatic neurocognitive impairment, Wang et al. integrated DTI and rs-fMRI data and proposed a white matter structure–function decoupling index to quantify the mismatch between microstructural integrity and functional activity, providing a new imaging perspective for the early detection of brain injury. In addition, Mu et al. combined cervical spinal cord DTI with thalamic magnetic resonance spectroscopy (MRS) to investigate the relationship between local microstructural injury and metabolic alterations in remote brain regions, highlighting the potential of multimodal MRI to reveal cross-regional neural changes.

## Dynamic functional organization reveals temporal and context-dependent brain alterations

Beyond conventional static functional connectivity analyses, several studies in this Research Topic further highlight the importance of characterizing brain functional organization across temporal, task-related, and frequency-specific dimensions. Xu et al. applied a hidden Markov model to rs-fMRI data from young patients with migraine without aura and identified abnormal transition patterns among dynamic functional connectivity states. These findings suggest that dynamic brain states and their temporal properties may provide information beyond that captured by conventional static connectivity analyses. Brain functional abnormalities may also become apparent during specific tasks or interventions. Yu et al. combined arterial spin labeling with resting-state functional connectivity analyses to investigate the immediate effects of acupuncture on brain function in patients with major depressive disorder. Their findings suggest that multimodal functional imaging can capture intervention-related changes in cerebral blood flow and network organization, offering a new perspective for evaluating the effects of neuromodulation. Chen J. et al. combined task-based fMRI and rs-fMRI to investigate audiovisual divided attention in people with HIV who did not yet meet the diagnostic criteria for HIV-associated neurocognitive disorders (HAND). They found evidence of functional network reorganization before the emergence of clinically apparent cognitive impairment, suggesting that task-related functional alterations may provide important clues to early brain injury. Mao et al. further examined resting-state brain activity across the Alzheimer's disease continuum from a frequency-specific perspective and found that spontaneous neural activity in different frequency bands showed distinct associations with diagnostic status and cognitive performance, underscoring the value of analyses across multiple temporal scales. Above all, these studies indicate that integrating dynamic connectivity, task-based functional assessment, and frequency-specific analyses can overcome some of the limitations of conventional static measures, provide a multidimensional characterization of disease-related functional network reorganization, and open new avenues for identifying clinically meaningful imaging biomarkers.

## From population differences to individualized phenotyping: biomarker discovery and clinical translation

Neuropsychiatric and neurological disorders exhibit substantial clinical and biological heterogeneity, which is not fully captured by conventional neuroimaging studies based primarily on group-level differences. The studies included in this Research Topic further explore how multimodal MRI, artificial intelligence, and clinical phenotypic information can be integrated to move beyond the identification of imaging abnormalities toward disease subtyping and individualized characterization. Casillas Martinez et al. integrated structural, diffusion, and functional MRI and identified distinct combinations of imaging features across different developmental stages of autism spectrum disorder, highlighting the importance of age stratification for establishing more precise imaging phenotypes. Pan et al. combined features of spatial functional organization with transcriptomic information to investigate associations between imaging abnormalities and their potential molecular underpinnings, providing a new perspective on linking macroscopic imaging phenotypes to underlying biological processes.

Artificial intelligence methods have further advanced neuroimaging research toward individualized subtyping and interpretable analysis. Zhang et al. used a graph-learning approach to integrate brain structural features with clinical phenotypes and identified potential subtypes among patients with insomnia disorder. Chen S. et al. highlighted the importance of reliable clinical assessment and patient stratification for improving both model performance and the biological interpretability of neuroimaging-based classification in disorders of consciousness. Yuan et al. proposed a knowledge-guided, interpretable artificial intelligence framework that incorporates neuroscientific knowledge into high-dimensional functional connectivity analysis, providing a new methodological approach for developing biologically informed imaging-based predictive models. In addition, Luan et al. proposed a research framework integrating interoceptive behavior, clinical symptoms, and cognitive measures with structural MRI, functional MRI, and DTI. This framework is designed to investigate potential relationships between the clinical manifestations of schizophrenia and abnormalities in relevant brain networks, offering a new approach to examining the neural basis of the disorder from the perspective of clinical phenotypes. Overall, these studies illustrate the ongoing transition of neuroimaging research from the description of group-level differences toward the development of individualized phenotypes, while advancing imaging biomarkers beyond disease identification toward mechanistic interpretation and clinical application.

Beyond methodological advances in neuroimaging, Jiang et al. systematically reviewed developments in immunological and neuroimaging research on HAND. They emphasized that isolated imaging abnormalities are insufficient to capture the complex mechanisms underlying the disease and advocated the integration of immune biomarkers, neuroinflammatory processes, and multimodal MRI features into a multilevel biomarker framework to support early detection and risk stratification.

## Conclusion

Overall, this Research Topic highlights the evolving role of multimodal MRI, from the integration of complementary imaging modalities and the characterization of brain networks to the development of individualized phenotypes. It also promotes closer integration of neuroimaging findings with clinical and biological data. Future multimodal imaging research should place greater emphasis on standardized analytical procedures, multicenter validation, and longitudinal follow-up to improve the reproducibility and clinical translatability of imaging biomarkers. By integrating advanced imaging techniques, artificial intelligence, and multidimensional biological information, neuroimaging research may progress beyond the description of associations toward mechanistic understanding and applications in precision medicine.

